# Impact of the armed conflict in Colombia: consequences in the health system, response and challenges

**DOI:** 10.1186/s13031-023-00561-6

**Published:** 2024-01-03

**Authors:** Oscar Bernal, Tatiana Garcia-Betancourt, Sebastián León-Giraldo, Lina Marcela Rodríguez, Catalina González-Uribe

**Affiliations:** 1 Alberto Lleras Camargo School of Government, Universidad de los Andes, Bogotá, 11711 Colombia; 2 School of Medicine, Universidad de los Andes, Bogotá, 11711 Colombia

**Keywords:** Armed conflict, Health systems, Policy, Colombia

## Abstract

**Introduction:**

In Colombia, research on health and conflict has focused on mental health, psychosocial care, displacement, morbidity, and mortality. Few scientific studies have assessed health system functioning during armed conflicts. In a new period characterized by the implementation of the peace agreement with the Revolutionary Armed Forces of Colombia (FARC) armed group, understanding the effects of armed conflict on the health system, the functions, and institutions shaped by the conflict is an opportunity to understand the pathways and scope of post-conflict health policy reforms. Therefore, this study was conducted to assess the effects of armed conflict on the health system, response, and mechanisms developed to protect medical missions during armed conflict in Colombia.

**Methods:**

This research was conducted using a qualitative approach with semi-structured interviews and focus group discussions. The qualitative guide collected information in four sections: (1) conflict and health system, effects and barriers in health service provision, (2) actions and coordination to cope with those barriers, (3) health policies and armed conflict, and (4) post-accord and current situation. Twenty-two people participated in the interviews, including eight policymakers at the national level and seven at the local level, including two NGOs and five members of international organizations. An academic project event in December 2019 and four focus groups were developed (World Cafe technique) to discuss with national and local stakeholders the effects of armed conflict on the health system and an analytical framework to analyze its consequences.

**Results:**

The conflict affected the health-seeking behavior of the population, limited access to healthcare provision, and affected health professionals, and was associated with inadequate medical supplies in conflict areas. The health system implemented mechanisms to protect the medical mission, regulate healthcare provision in conflict areas, and commit to healthcare provision (mental and physical health services) for the population displaced by conflict.

**Conclusion:**

The state’s presence, trust, and legitimacy have significantly reduced in recent years. However, it is crucial to restore them by ensuring that state and health services are physically present in all territories, including remote and rural areas.

## Background

The direct effects of armed conflict on health have been well documented in the literature; mortality, morbidity, and disability due to different causes are some of the effects assessed in the short-, medium-, and long-term [1, 2]. Population, environment, and state structures are part of those dimensions directly affected by armed conflict and war. The functioning of health systems, policy formulation and implementation, management, resources (material, financial, and human resources), and service delivery are elements that are affected during armed conflict [3]. Events including the destruction of clinics and hospital infrastructure, loss of health workers, medical treatment interruptions due to unavailability, and difficult access to health facilities are global concerns [4].

Colombian armed conflict, actors, victims, and territories are heterogeneous [5]. Different forms of violence have been seen, including combats, bombing, attacks on the population, selective murder, massacre, kidnapping, forced disappearance, recruitment, and damage to civil property [6]. The conflict caused approximately 220,000 deaths (1958–2012), 1,982 massacres (1980–2012), 27,023 kidnappings (1970–2010), 25,007 missing, and more than 4,744,046 displaced people [7].

The country is currently facing a new period characterized by the implementation of the peace agreement with the FARC (Revolutionary Armed Forces of Colombia) armed group, signed on November 24, 2016 [8]. After four years of negotiations, the “Final Agreement for the End of Conflict and the Construction of a Stable and Lasting Peace’ was signed, which focuses on five aspects: comprehensive rural reform, political participation, end of the conflict, illicit drugs and truth, justice, reparation, and non-repetition [9]. However, implementing the peace agreement has been challenged by the murder of community leaders, attacks on opposition political organizations, reactivated paramilitary groups, and some dissident armed groups and a new government with different approaches to conflict management. One of the most ambitious proposals of the government of Gustavo Petro is the concept of ‘total peace,’ in which he aims to negotiate with as many armed groups as possible during his four years in office to end the conflict in the country. These include the National Liberation Army (ELN), FARC dissidents, and criminal gangs like the Clan del Golfo. In this framework, benefits such as reduced sentences and non-extradition would be in exchange for surrendering weapons, providing information on drug trafficking routes, and delivering goods and money resulting from illegal activities [10].

A society transitioning towards peace has challenges and should respond to multiple demands and anticipate new scenarios [11]. The health system in Colombia, as a structure that guarantees health as a right and organizes, provides, and finances the different health services, is a fundamental element in this conjecture [11]. Understanding the effects of armed conflict on the health system and the functions and institutions shaped by conflict is an opportunity to understand the pathways and scope of post-conflict health policy reforms.

Few studies have assessed health system functioning during armed conflict. In Colombia, research on health and conflict has focused on mental health [12–16], psychosocial attention [17, 18], displacement [19–21], mobility, and mortality [22, 23]. Access to health services [20] by displaced populations, the impact of armed groups on affiliation coverage [24], vaccination coverage [22], and costs related to armed conflict [22] are some of the studies conducted in Colombia.

This study assesses the consequences on the population and health system, the response and challenges of the health system during armed conflict, and post-agreement in Colombia.

## Methods

A qualitative study using Ground Theory methodology and the following steps: transcribe interviews and review a small sample of text, identify potential analytic categories (themes) that arise as the categories emerge, pull together all the data from those categories and compare them, consider how categories are interconnected, use the relations among categories to build theoretical models, and present the results of the analysis using quotes from the interviews that illuminate the theory (exemplars).

A qualitative study was conducted through semi-structured interviews (Annex 1, Interview Guide). A total of twenty-two people were interviewed: eight policymakers at the national level (Ministry of Health officials), seven at the local level (officials of local secretaries of Health), two NGOs (such as the Associated Corporate Foundations), and five members of international organizations (Such as the Red Cross, Organization for Migration (IOM), Doctors Without Borders, United Nations Population Fund, and the Pan American Health Organization).

Data collection was conducted principally in Bogotá, Colombia, from September 2019 to May 2020. Four focus groups were developed during an academic project event in December 2019 (Coffee World technique). The main objective was to discuss with national and local stakeholders the effects of armed conflict on the health system and an analytical framework to analyze its consequences. The interview and focus group data were transcribed and analyzed using NVIVO 12 software (QSR International) for coding.

The analysis used pre-specified codes based on emerging categories from the information collected: (1) conflict and health system; effects and barriers in health service provision; for this category, the WHO framework for health system was used to cluster the consequences. (2) actions and coordination to cope with those barriers; (3) health policies and armed conflict; design, adaptation, and implementation of health policies.

## Results

### Direct and indirect consequences

The main direct impacts on the health of the population included the death of combatants and civilians; the disability that occurs second to injuries in combat, explosive devices, and mines; the sexual violence of which mainly women were victims; and mental health disorders.

*“There are many types of health effects, let’s say there are direct ones, those that have to do with the physical and mental affectation left by the armed confrontation, or the traumatic events derived from the armed confrontation, such as forced displacement. There are also a series of indirect effects that I think are sometimes not considered important, but which are crucial, for example, the effects it has on health and the quality of life and water, the blowing up of oil pipelines in Nariño, Catatumbo, and Arauca. Or think about how illegal mining leads to the development of water ponds like Disneyland for malaria or, for example, the nutritional health effects on populations confined by armed confrontation. Violence also affects this type of flow; it also affects the flow of medicines”* (Public National Sector, 2019).

Indirect effects are associated with morbidity from other causes, not directly from the armed conflict, but from the context of the conflict, such as the lack of essential services, vector-borne diseases, and lack of care and access to treatment of chronic illnesses.

A key element highlighted in the previous quote is the confinement situation, which has both direct and indirect effects on the health of civilian populations. The effects on nutritional health, food, and drug distribution are also mentioned.

*“In Colombia, I was very favorably surprised that the confinements were identified as important official events; this does not happen in any other country in the world or in humanitarian literature. The one who is seen is the one who flees; the displaced but the confined are not there, and I think that the confinements that I had always thought of as a besieged city due to normal warfare have a terrible impact on this what is happening in Chocó and others- which is not measured either”* (Private National Sector 2020).

### Consequences on the health system

#### Leadership, governance, and provision of services

In the zones most affected by armed conflict, both due to the strong presence of armed groups and confinement issues, leadership and governance were under the control of the armed groups or exercised at the local level. The latter, in addition to the limited presence of the state in its different structures, directly affected the poor provision of health and care services.

*“The populations that were in conflict were isolated from the institutions; what the institutions did was to leave the countryside and not risk going into zones where government participation was impeded (…) In the conflict, this affects people. It is a deployment of the armed groups, and the ones who were there were the ones who provided medical assistance, and the others, the institutionality was deployed.”* (Working Table 2020).

*“The regions, especially in the conflict, had a total division with the presence of the government, despite there being a municipality, despite there being a mayor, there’s a total loss of democracy; I see it as these regions having some dynamics that the mafia cultures, be it the coca-growing-culture of one year or another, be it the guerrillas-culture, or the paramilitary-culture, took control of the state at the local level, in one way or another, either in a subtle way or in a very aggressive way within the conflict.”* (NGO 2019).

This type of governance depends on the interests of in-office leaders at any given time and the local response capacity, which reinforces the differences among territories and their response to health problems depending on the existing service network, cooperation with other institutions, and internal dynamics at the local and regional levels.

*“The health sector has a characteristic, and it is that all public health services are executed at the municipal level, and at the national level, there are programs; these programs at the municipal level provide the same actions that the public health plan should provide, the city of Bogotá, the municipality of Rosas in Cauca, the same, or the municipality of Dibulla over there in La Guajira, have the mayor that they have, have the same functions in public health, but the difference in resources is abysmal, inhuman, technical resources, and in implementation capacity (…).”* (National Health Sector 2019).

#### Health system financing

Government priorities act as factors in budget and resource allocation. During the more than 50 years in Colombia, these priorities have changed, which has affected resources and financing for health.

*“The very dynamics of the conflict make public resources go away; it makes it so that it is preferred to allocate them to security, to the army, and that limits or diminishes the resources available for health, so undoubtedly when the national budget is shown, and it is seen that there is a great proportion of resources allocated for the war, to finance the war, for the military forces, in the end, that money is no longer being used in other strategies, including health, although it is challenging to know what would have been done if we were not at war”* (National Public Sector, 2019).

Specifically, in health activities, institutional limitations and limited government access to territories affected the financing of health programs. Mainly, the high costs of transportation to reach conflict zones are identified for professionals, services, treatments, and even health promotion and prevention activities, budget adjustments to carry out health activities, the limited availability of personnel, and the increase in the cost of having health professionals in the rural sector. The latter is in addition to structural barriers such as corruption and limited budget control efforts in areas where institutionality is limited.

*“In terms of costs, health costs skyrocketed; there are at least three reasons: number one, the very cost of processing care (unintelligible); number two, the cost of providing services in areas where there is violence is higher because this implies greater expenses in security; and number three, in some places in the country health professionals, need to be paid more so that they want to go there. The armed conflict overlaps with great frequency, so, for example, in some areas, there were acts of corruption in favor of armed groups who wanted health resources to use them in financing war weapons”* (Working Table 2020).

#### Health human resources

The shortage or total lack of health personnel is caused by insecurity in different territories. The murders, kidnappings, and attacks on medical missions caused insufficient personnel to provide health care in many territories.

*“When there was a conflict, it was impossible to get there either because they did not give permission or because they had to ask for permission or risk that the medical team would be retained, because it happened on several occasions, they retained, because then they would not only go there to attend to the peasant population who was in some way affiliated with a health service provider or who was in charge of the very State, but they ended up in the hands of the insurgent groups who took the doctors to attend to their personnel, who were, at that time, with a health problem. Thus, that generated fear concern, and the doctors no longer wanted to go out to the health brigades, neither did the dentist; that is, people were afraid to go to the territory, to the rural sector.”* (Public Health Department 2020).

In cases where care services were continued, fear of personal safety was permanent. Health professionals were kidnapped for the health care of the armed groups, and combats and violent attacks on health facilities were conducted.

*“One, the fear of one as a doctor of knowing whom you are treating, then a wounded person would arrive, and one cannot ask, and at any moment other insurgents would arrive to kill him inside the health center, then one does the human part without knowing who the person is, that ethical part, you cannot get into that, but you have to take care of a human life without thinking who it is, if it is bad or good, and that social pressure to be treated or to do something; aside from that, going out to the communities and many doctors being kidnapped, when I was there they kidnapped a colleague of mine because there was at that time a person who was injured from the guerrilla so they kidnapped him so that he could be there with them while he recovered.”* (Public Health Department 2020).

On the other hand, it was identified that there are structural barriers related to providing services and human resources, which exacerbate conflict. However, they are due greatly to the system’s configuration, such as medical professionals in rural areas, health education, and working conditions.

*“I think that the conflict is one of the strongest variables why people do not want to go to the territory, but I think that I repeat, if you eliminate the conflict variable, would there be more people who would want to go to the territory? Due to the problem of the conditions of the people, of the rural doctor, with up to 140 hours a week of attention, eh, with large investments in their careers, because studying medicine here is not cheap, in very few universities you can do it publicly, with meager rates of return on investment in their careers, when they go out into the territory and with training that increasingly devalues the decision-making capacities and responsibilities of health practitioners. Thus, I believe the conflict makes the problem more pronounced, but if you remove the conflict variable, survey how many doctors would go to the territory …”* (Working Table, 2020).

#### Medical products, vaccines, and technologies

Owing to a lack of accessibility or confinement, medical products and technologies were absent in some territories, which led to a total lack of medicines and diagnostic resources. Specifically, glucantime (a drug for leishmaniasis) has been reported as a medical product that directly participates in the dynamics of armed conflicts owing to its association with combatants. Access to controlled medicine and information on cases were associated with the context of armed conflict.

*“Regarding medicine, what happens with drugs against leishmaniasis, which were used as a weapon of war? Leishmaniasis is a problem, especially in rural areas; therefore, the state restricts the sale and use of this drug. It was impossible to obtain (…) Then, to determine if the patient was a member of the guerrilla groups and to limit access to many people who, regardless of whether or not they were outside of the law, needed treatment.* (National Public Sector, 2019).

Regarding infrastructure, interviewees reported the misuse of available resources during armed conflicts. Many constructions have been made based on local income/royalties from mining and oil exploitation. These buildings were sometimes underutilized because of armed conflicts or political issues.

*“Yes, in infrastructure, and I am going to talk about the particular situation of the department of Meta, there is an interesting infrastructure built during several governments, but that infrastructure is, on the one hand, a little over-dimensioned and on the other hand underused. That is to say, there are many of those infrastructures whose degree of occupation does not exceed 10%, and then several issues come into play, the political issue and the issue of the conflict, because, as I was telling you when people cannot move towards the municipality where the health center is located, then this generates underutilization.”* (Departmental Private Sector 2020).

#### Health information systems

As mentioned in the case of glucan time, the recording of patient data in conflict zones was a barrier, which led to a complete lack of information on specific diseases or a shortage of data from some territories in Colombia. Additionally, in confined zones, there were no medical records of the population or monitoring of the health situation. The elements with the most challenges for notification were those related to the dynamics of conflict, such as sexual violence and combat-associated diseases.

*“We don’t even know what happened in the conflict; several victims still have not been able to be reported (…) Alternatively, there are isolated populations, where no one knows anything about what is happening. (Participant 2) I think it was Pandora’s box. It is opened, and many things begin to be discovered; the threat against life implies the need for silence (…)*.

*Yes, yes, I want to mention one that has to do with the medical mission, and it is, in general, the health agents in the places where we go always tell us, “Hey, please ask the obligation of reporting cases of violence to the police to be removed from us, that obligation puts us in trouble.”* (Working Table 2020).

### Health system response

The response of the health system and other organizations at the local and national levels consists of adapting the protocol and applying regulations to specific territories.

These activities during armed conflicts were based on intersectoral initiatives for the performance of NGOs and other organizations. It is essential to highlight that the response differs depending on the local capacities, context, history, and resources.

*“The preparation for the response did not occur; some things were done through international support; the United Nations and all those organizations responded more to the conflict than the country itself. From the first years of 2000, when violence was resurgent with acts of great force, the country looked at them, but there was no response from the specific sector; in the post-conflict period, let’s say that work has begun.”* (Departmental Public Health Entity 2020).

A key element identified in the interviews was the delimitation and clarification of armed conflict in designing public policies. This issue relates to the peace agreement of the FARC group as a milestone in public policy. Therefore, the first step was the national consensus and definition of armed conflict, the victims, and the prioritization of the national agendas.

*“What one saw about the armed conflict is that, first, all the [discussion] that existed and still exists on whether the conflict exists or not, and that from the central state itself, is a limitation for the generation of policies, if even some voices continue to say that there is no armed conflict, that there are other social problems, that makes it difficult, however, there are laws and the one that we can highlight is, effectively, the victims’ law.”* (National Public Sector 2019).

Some public policies have been designed and applied based on armed conflicts. Among these, the following stood out during the interviews and the worktables: PAPSIVI (Psychosocial and Comprehensive Health Program for Victims), mental health policies, and the Rural Health Plan of the Peace Agreement.

Difficulties in implementing these policies are based on the lack of human resources at all levels, lack of coordination at the local level, corruption, and shallow or volatile stakeholder interest in the implementation and oversight of the process.

*“When one is outside the Ministry, one says, but why doesn’t the Ministry act? Right? Where is the State? However, when you see the Ministry, you realize that it is the governing body. Still, we do not lend there; we are not there, so we make some rules, some administrative acts, eh, that have multiple levels and that intend to reach the end user, towards the final individual, and that is not as easy to implement as you think, right? The processes are not so fast, even from when we issue until it reaches the last official or individual; it may take years.”* (National Public Sector 2019).

*“We still have to systematize even more. From a political perspective, it is also difficult to commit territories to a bet with a vision of 15 years in the long term. You know, we are not doing the planning very well (…) I think that one of the challenges, and in general a challenge of peace processes, is that sustainability depends on showing results in the short term, so there is a lot of pressure for quick results. it is good and has to be done, but that is a generic problem of the public sector: we are eternally trapped in the situation.”* (National Public Sector 2019).

#### Does this problem lie in the insurance model?

Several interviewees pointed out that the problem of access and quality of care is due to the insurance and financial intermediation model, considering that a change in the insurance structure is required, not only to respond to the conflict but also to multiple needs, especially in populations living in dispersed rural areas.

*“It seems to me that this discourse does not align with reality. Our general social security system did not respond to our conflicts, nor did it have the capacity to rise (unintelligible) as primary care. The general system does not consider differential care for the victims. It does not think about it because our system is an economic system and transaction. Suppose you hire a health service provider to visit the population. In that case, the end-provider does only what the health service provider told them, and that is it, so the discourse of primary care is a reality among us who have lived it for many years, but Law 100 ended it. Therefore, the system must be modified accordingly. Attention must be paid to areas, especially dispersed ones, rural, complicated, and a system that is not an economic transaction, without making an economic transaction but doing what it has to do, and there has to be another way. Trainers must train human resources for this diverse country, full of complex situations, and start again with values and ethics. The humanization of service, to respond not only to unbalanced people but to our community that lives in tough conditions, on the banks of the rivers.”* (Working Table 2020).

## Discussion

The Colombian health system has distinct characteristics that significantly influenced our findings. Colombian healthcare relies on a dual structure. One part consists of contributions from the insured population, while the other is bolstered by government subsidies, which are directed towards the subsidized regime, benefiting approximately 50% of the population [25]. On the other hand, Colombia has a mix of public and private healthcare facilities. While private institutions typically possess more resources and advanced equipment, public healthcare facilities, especially in rural regions, face challenges owing to limited funding [24]. This discrepancy can lead to considerable disparities in both the quality of care and accessibility to healthcare services.

Furthermore, Colombia is home to a commendable number of healthcare professionals, which, prima facie, suggests ample healthcare coverage [25]. However, this statistic is skewed by the pronounced concentration of these professionals in the urban areas. This urban-centric distribution exacerbates healthcare disparities as rural regions often find themselves underserved and lack adequate medical expertise and facilities.

Armed conflict has directly and indirectly affected the population and the Colombian health system and its ability to guarantee access and quality of health services, especially in dispersed rural areas [26–28]. The institutional response is affected by the limited presence of the state in conflict-affected regions, limited capacity at the local level, and the structure of the insurance system [28, 29].

Similar to what was identified in Yemen [30] and North Syria [31], this study identifies direct effects on the health system, such as attacks on health infrastructure [30], the lack of provision of health services [31], challenges in providing care related to territorial inequities between urban and rural areas, and drug distribution barriers [28–32], which have been elements present in the literature on the consequences of armed conflict on the functioning of the health system.

This conflict implies a reduction in or total lack of the provision of health services in some areas of the country, the loss of personnel, and a void in the presence of the state structure, as has been identified in this study. In 2019, attacks against medical missions were the highest recorded in the last four years, of which 56% were attacks carried out by patients, family members, and community members [33, 34], which indicates a change in violence against medical personnel that must be treated within the framework of the provision of services.

Similarly, this study highlights the importance of generating change and incentives within the peace agreement framework to provide services in rural areas. Among the elements found in the literature, several have been identified: the importance of understanding the supply of health personnel, education and training processes, salary system and personnel selection, distribution of the workforce, and incentive policies that address the problem of geographical imbalances in the labor force [35, 36].

This study portrays the dynamics of confinement as a direct effect related to the presence of the state in these areas, leadership, and governance. In 2019, 27,694 confined people were reported in the departments of Chocó, Antioquia, Nariño, and Putumayo [33]. In Colombia, the dynamics of confinement have been evidenced by organizations such as “Defensoría del pueblo” (Ombudsman’s Office), judicial verdicts, and decrees [37–39]. It is important to understand the historical dynamics of the confined population and their direct and indirect consequences as well as the necessary strategies and interventions.

In the last decade, progress has been documented in formulating public policies, plans such as the Ten-Year Public Health Plan and Comprehensive Health Care Policy, methodological guides, strategic guidelines, and the inclusion of differential approaches in the territory [40]. According to the World Bank Group (WBG), progress in the quality of care in Colombia has been documented, related to the expansion of Universal Health Coverage and improvement in user satisfaction [41]. According to an OECD study in 2016, Colombia has made remarkable progress in coverage, decreased out-of-pocket spending, reductions in unmet health needs, reduced waiting times for appointments, increased preventive health care visits, and increased perception of quality by service users [42].

In contrast to the advances described above, negative perceptions and challenges were identified for the sector. These include the problems of financial sustainability and little control of the prices of services [42, 43], increased costs due to duplication of services, excessive use of available resources, and low comprehensiveness of care [42], the strengthening of quality strategies at the national level and their alienation from the Health Promoting Entities, the health authorities at the departmental level, and the health care providers [42]. In addition to these challenges, the current model of care, fragmentation, and the need for effective integration and coordination of care are also important [42]. It has been identified that the system of financial incentives distributed to multiple actors, which is related to high administrative decentralization, creates access barriers for users [29, 42, 43]; this adds to the problematic coordination and deficiencies in communication between primary care and specialized services [44].

It is important to note that while this study had a limited sample size and did not include participants from the general population, the interviewees were experts and decision-makers in their field, who were also stakeholders. Although their perspectives may have included those of the general population, it is crucial to acknowledge that they may have been biased based on political preferences.

## Conclusions

As identified in this study, the state’s presence, trust, and legitimacy have been truncated during these years concerning armed conflict and due to different governance problems such as technical capacity and corruption cases. Restoring legitimacy is crucial and should be based on the real presence of state and health services in territories and dispersed rural areas.

In this context, attacks on medical missions have continued to increase. This situation calls for dialogue with communities and policies to reduce barriers to accessing health services, particularly in remote rural areas. Such policies should be designed to prevent the isolation of communities and to ensure genuine access to healthcare services.

Health sector financing needs to prioritize the quality and accessibility of services, particularly for communities affected by conflicts. Healthcare providers must be able to work without interference from the armed groups. Policies must be tailored to meet the needs of the population, and local resources and capacities must be strengthened to implement, evaluate, and monitor these policies effectively.

## Data Availability

The datasets generated during the current study are not publicly available because they contain information that could compromise research participant privacy/consent, but are available from the corresponding author upon reasonable request.
